# Overexpression of Activating Transcription Factor-2 (ATF-2) Activates Wnt/Ca^2+^ Signaling Pathways and Promotes Proliferation and Invasion in Non-Small-Cell Lung Cancer

**DOI:** 10.1155/2022/5772089

**Published:** 2022-06-02

**Authors:** Li Zhang, Shanggan Zeng, Zhanzheng Yu, Guangxing Zhang, Zhenfang Xiong, Fuyuan Xie, Zhenyu You

**Affiliations:** ^1^Department of Thoracic Surgery, The First Affiliated Hospital of Nanchang University, Nanchang, China; ^2^Department of Oncology, The First Affiliated Hospital of Nanchang University, Nanchang, China; ^3^Department of Pathology, The First Affiliated Hospital of Nanchang University, Nanchang, China

## Abstract

Previous studies have suggested an association of the expression of activating transcription factor-2 (ATF-2) with the survival time and the activity of the Wnt/Ca^2+^ signaling pathway in non-small-cell lung cancer (NSCLC). However, the exact role of ATF-2 in tumorigenesis and its underlying mechanism remains unclear. In this study, we study whether ATF-2 regulates the growth and reproduction of NSCLC cells through the Wnt/Ca^2+^ pathway. The expression of ATF-2 and pathway-related genes in non-small-cell lung cancer was detected by qRT-PCR and Western blotting. CRISPR/Cas9 technology was used to knock out the ATF-2 gene, and pathway inhibitors and agonists were added to induce cultured cells. The expression of pathway genes and the proliferation and invasion ability of A549 lung cancer cells were analyzed. ATF-2 and pathway-related genes were upregulated in NSCLC. The proliferation and invasion ability of A549 lung cancer cells was decreased after only adding pathway inhibitors. The expression of Wnt/Ca^2+^ pathway protein was decreased when the ATF-2 gene was knocked out, but the expression of Wnt/Ca^2+^ pathway protein was reversed after the addition of a pathway agonist. These results suggest that ATF-2 acts as an agonist in the Wnt/Ca^2+^ signaling pathway, promoting the expression of Wnt5a, Wnt11, CaMK II, and NLK in the Wnt/Ca^2+^ pathway, thereby regulating the proliferation and invasion of NSCLC cells.

## 1. Introduction

Lung cancer is one of the most common malignant tumors in the world today, and it is a serious threat to human life and health, with more than 1 million people dying of lung cancer every year [1]. Non-small-cell lung cancer (NSCLC) is the most common type of lung cancer, accounting for about 80% to 85% of lung cancer. Due to the lack of early diagnosis, more than two-thirds of patients are diagnosed at an advanced stage when they are found. Advanced NSCLC is treated with combination therapy mainly with chemotherapy, but the effective rate of chemotherapy is 30%-50% [2]. With the deepening of the research in recent years, the research on lung cancer has made a lot of achievements, but drug resistance, recurrence, metastasis, and other problems after treatment are still a huge obstacle to the long-term survival of lung cancer patients. Therefore, for the early diagnosis and precise treatment of lung cancer, it is of theoretical and clinical significance to analyze the mechanism of the occurrence and development of lung cancer at the molecular level and find the genes closely related to the occurrence of lung cancer.

During the occurrence and development of NSCLC, multiple signaling pathways play a very important role, including Hh signaling pathway [3] and Wnt signaling pathway [4, 5]. Wnt is a family of protooncogenes, and the first Wnt gene was cloned from a mouse genome in 1982 [6]. In the following years, several Wnt genes were isolated from different species, and their biological functions gradually attracted wide attention. The Wnt signaling pathway is highly conserved in evolution. It is cooperated by Wnt signaling proteins, Frizzled receptor proteins across the membrane, and various cytoplasm proteins within the cells, transmitting signals from the cell surface to the nucleus. Currently, it is believed that Wnt conducts through three main pathways [7, 8]: (1) Wnt/*β*-catenin pathway, (2) Wnt/polarity pathway, and (3) Wnt/Ca^2+^ pathway. *β*-Catenin is a key factor in the canonical Wnt pathway, transmitting signals into the nucleus and interacting with the transcription factor LEF/TCF to activate the expression of protooncogenes. Survival curve analysis showed that the expression levels of Wnt and *β*-catenin were significantly negatively correlated with survival rate of patients [9].

With further research on the Wnt/*β*-catenin pathway in NSCLC, the role of the Wnt/Ca^2+^ pathway in NSCLC has gradually attracted more attention. In the Wnt/Ca^2+^ pathway, many factors are involved in signal transmission, such as Wnt5a and Wnt11. In the Wnt/Ca^2+^ signaling pathway, the Wnt5a gene has been shown to activate the calcic-dependent signaling pathway to stimulate cancer invasion and epithelial-mesenchymal transformation [10, 11]. Studies have shown that Wnt7a and Frizzled can induce the expression of calcadherin protein, inhibit the epithelial differentiation of NSCLC cell lines, and promote the EMT process of NSCLC cells [12, 13]. The Wnt pathway can regulate the occurrence and development of NSCLC, which makes the Wnt/Ca^2+^ signaling pathway have the potential to be a drug target for the treatment of NSCLC.

Activating transcription factor-2 (ATF-2) belongs to the transcription factor family of ATF/CREB (cAMP response element binding protein). It is an important component of the activator protein-1 (AP-1) transcription complex [14]. ATF-2 plays an important role in the regulation of normal cell growth and development, stress response, DNA damage response, and other links [14, 15]. However, the role of ATF-2 in tumor cell growth is still unclear. ATF-2 knockdown in melanoma cells can increase c-Jun expression and promote apoptosis [16]. Downregulation of ATF-2 expression significantly inhibited the migration ability of HepG2 and SK-Hep-1 cells [17]. In breast cancer patients, high expression of ATF-2 is positively correlated with the survival of patients, while patients with low expression of ATF-2 have worse postoperative effects [18]. In conclusion, ATF-2 is a molecule with protocancer and anticancer functional activity and has different functions in different tumors. To date, the role of ATF-2 in NSCLC is poorly understood. Studies have shown that there is a negative correlation between expression of ATF-2 and survival time of lung cancer patients; that is, patients with high expression of ATF-2 have a short survival time, while patients with low expression of ATF-2 have a long survival time [19]. Therefore, it is speculated that the expression of ATF-2 can promote the proliferation and development of lung cancer cells. However, the mechanism by which ATF-2 promotes the growth of NSCLC is still unclear. Through luciferase reporter system analysis, we found that ATF-2 can activate the Wnt/Ca^2+^ signaling pathway and further regulate embryonic development and the development of NSCLC [20, 21].

Therefore, this paper explores whether ATF-2 promotes the development of NSCLC by activating Wnt/Ca^2+^ signaling pathways. First, the expression of ATF-2- and Wnt/Ca^2+^-related proteins in NSCLC was detected. XAV939 was added to inhibit the activity of the Wnt signaling pathway, and the effect of ATF-2 on the Wnt/Ca^2+^ signaling pathway was verified. XAV939 inhibits pathway activity by promoting the degradation of Wnt pathway-related genes.

## 2. Materials and Methods

### 2.1. Cell Culture

A normal bronchial epithelial cell line (HBE) was derived from Shanghai Ysribio Industrial Co., Ltd. Human lung adenocarcinoma adjacent normal cells and human lung cancer cell lines PC9, H1650, H1299, and H446 were all from the First Affiliated Hospital of Nanchang University. The sources of cells used in this study have obtained the informed consent of patients and the approval of the First Affiliated Hospital of Nanchang University Ethics Committee.

All the cells and cell lines were cultured in the medium supplemented with 10% fetal bovine serum, 100 mg/mL streptomycin, and 100 U/mL penicillin and cultured in an incubator at 37°C and 5% CO_2_. The cells were multiplied to a certain number for reserve use.

A549 cells in a logarithmic growth phase were seeded into 96-well plates and then added a pathway inhibitor (XAV939) (AbMole, US) or agonist (LiCl) (Sigma-Aldrich, Merck) to induce culture.

### 2.2. qRT-PCR

Total RNA in cells or tissues was extracted, and cDNA was obtained by reverse transcription with PolyA primer and Invitrogen™ reverse transcription kit (Thermo Fisher Scientific). Using cDNA as a template and U6 as an internal reference, PCR was performed for the target gene. The mRNA levels of specific genes were detected on a quantitative PCR instrument, and three duplicate holes were set in each sample for miRNA detection to reduce errors. The primers used are as follows: ATF-2: forward 5′-TGGTAGCGGATTGGTTAGG-3′ and reverse 5′-TTGGGTCTGTGGAGTTGTG-3′; Wnt5a: forward 5′-CAGTTCAAGACCGTGCAGAC-3′ and reverse 5′-TGGAACCTACCCATCCCATA-3′; Wnt11: forward 5′-TGACCTCAAGACCCGATACC-3′ and reverse 5′-CAAGTGAAGGCAAAGCACAA3; CaMK II: forward 5′-CCAGCCACTGTATACATCAGAT-3′ and reverse 5′-CACAGGTTTTCCATAGGGATCT-3′; NLK: forward: 5′-AGGCTCCTGAGAATCAACCCAAC-3′ and reverse 5′-CCACGGTAATTGACCAACCTCTG-3′; LEF1: forward 5′-TTATCCAGGCTGGTCTGCAAGAG-3′ and reverse: 5′-GCAGCTGTCATTCTTGGACCTGTA-3′; TCF1: forward 5′-GAACTGGCCAAGCTGAGGTG-3′ and reverse 5′-GAGGCTTCTGAGTGTTAGTCCTGTC-3′; and GAPDH: forward 5′-TGGATCAGCAAGCAGCAGGAGTA-3′ and reverse 5′-TCGGCCACATTGTGTGAACTTT-3′.

### 2.3. Western Blot Assay

The cells and tissues to be tested were collected; then, the cells or tissues were lysed with NP-40 cell lysate, and the supernatant was extracted by centrifugation. Add an equal volume of 2× SDS loading buffer to the supernatant, and boil for 5 minutes to denature the protein. SDS-PAGE was used for electrophoresis, and the cellulose acetate membrane was used for a constant pressure transfer membrane. A primary antibody was added to the membrane and placed in an incubator for overnight incubation at 4°C and then washed three times with PBS for 10 minutes each time. Add a sheep anti-rabbit IgG antibody (1 : 10000, Cell Signaling Technology) and incubate at room temperature for 2 hours. Wash with PBS three times for 10 minutes each time. Finally, the film is pressed and developed. The antibodies used in this experiment include ATF-2 antibodies, Wnt5a antibodies, Wnt11 antibodies, CaMK II antibodies, NLK antibodies, TCF1 antibodies (1 : 1000, Amyjet Scientific, Wuhan), and *β*-actin antibodies (1 : 2000, Abcam).

### 2.4. Immunohistochemical

NSCLC and paracancerous tissues were taken and embedded in paraffin, followed by paraffin section. Sections were fixed with paraformaldehyde at room temperature for 20 minutes and rinsed with PBS for 3 times. Add 5% BSA at room temperature and seal for 20 minutes; then, add an ATF-2 antibody (FineTest®, China) and incubate overnight in a refrigerator at 4°C. Rinse with PBS for 3 times, add appropriate concentration of an HRP conjugated secondary antibody (Wolcavi Biotech, Beijing, China), and incubate at room temperature for 2 hours. Rinse with PBS 3 times for 10 minutes each time. The nuclear stain DAPI (Sigma-Aldrich, Merck) was added and dyed at room temperature for 10 minutes without light. The excess DAPI was washed with PBS. Drops of 80% glycerin were added to the slide, and the cover glass was used to seal the slide. Finally, the slide was observed with a fluorescence microscope and photographed.

### 2.5. CRISPR/Cas9 Technology

Using the CHOPCHOP online tools (http://chopchop.cbu.uib.no/), design for the gRNA ATF-2 gene (TTCAGTCCTCACCCAGATGGCGG) both ends with sticky end. By denaturing annealing, gRNA was linked to the skeleton carrier (Addgene) to obtain pSpCas9 (BB)-2A-GFP-gRNA plasmid [22]. The plasmid was transfected into A549 cells by Lipo2000 (Sigma-Aldrich, Merck); after 24 h of culture, the cells carrying GFP were selected by flow cytometry (Beckman Coulter), that is, the transfected plasmid cells. The selected cells were inoculated into 96-well plates for single cell culture (one cell per well) for several days. After the number of cells increased, part of the cell genome DNA was extracted and identified by PCR and Sanger sequencing, and the cells with frameshift mutations were obtained, which were the cells needed. ATF-2 knockout efficiency was further confirmed by WB.

### 2.6. CCK8 Assay

The transfected cells were collected, placed on a 96-well plate to prepare a suspension, and cultured in an incubator at 37°C under 5% CO_2_ for 24 h. 10 *μ*L 10% CCK8 solution was added, and the culture was continued at 37°C for 2~3 h with 5% CO_2_. OD_450_ was determined on the enzyme plate analyzer (Perlong).

### 2.7. Transwell Night Assay

Human NSCLC cells in a logarithmic growth phase were inoculated into 6-well plates after digestion with trypsin and given different interventions (transfection of different plasmids or adding of different pathway regulating reagents to induce culture). The Transwell chamber and 24-well plate were precooled, and Matrigel glue was spread. Trypsin digestion, cell count, and adjustment of NSCLC cell density were performed. The cells were inoculated with MEM containing 500 *μ*L 10% FBS in the lower chamber of the Transwell chamber. And cell suspension 200 *μ*L was added into the Transwell chamber for conventional culture for 48 h. 4% formaldehyde was fixed for 15 minutes, Giemsa dye (Sigma-Aldrich, Merck) was stained for 25 minutes, PBS was rinsed twice, and a microscope was used for observation.

### 2.8. Statistics

SPSS 25.0 software was used for data analysis in this experiment, and the data were expressed as mean ± standard deviation ($\bar{x}\pm s$). The *t*-test was used for comparison between the two groups, and *P* < 0.05 was considered statistically significant. The data were processed by GraphPad 8.0 software and presented in the form of charts.

## 3. Results

### 3.1. ATF-2 Expression Level and Wnt/Ca^2+^ Signaling Pathway Activity in NSCLC

**Figure 1:**
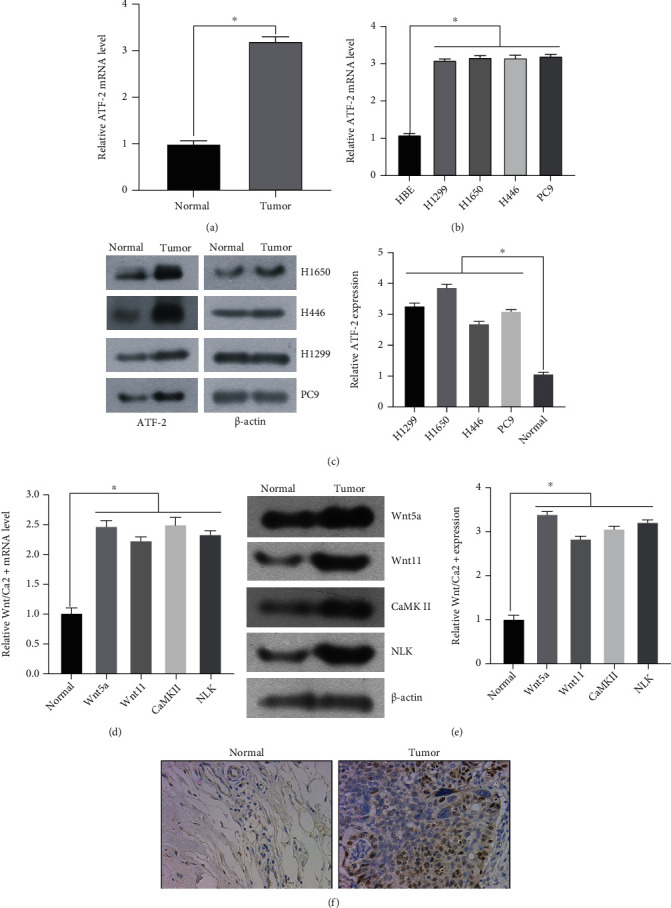
The expression of ATF-2 and Wnt pathway proteins in lung cancer tissues and cells was detected. (a–c) The expression of ATF-2 in lung cancer cells and HBE was detected by qPCR and WB. (d, e) qPCR and WB were used to detect the expression of Wnt pathway proteins in lung cancer cells and normal cells. (f) The expression of ATF-2 in lung cancer cells was detected by IHC.

The expression of ATF-2 in normal bronchial epithelial cells, non-small-cell lung cancer tissues, and paracancerous normal tissues was detected by qRT-PCR. The qRT-PCR results are shown in Figures 1(a) and 1(b). ATF-2 is overexpressed in non-small-cell lung cancer tissues. The expressions of Wnt/Ca^2+^ pathway proteins in NSCLC tissues and adjacent normal tissues were detected by qRT-PCR. qRT-PCR results showed that the pathway proteins Wnt5a, Wnt11, CaMK II, and NLK were upregulated in NSCLC tissues (Figure 1(d)). WB test results showed that ATF-2 and Wnt/Ca^2+^ pathway proteins were highly expressed in NSCLC tissues (Figures 1(c) and 1(e)). The expression of ATF-2 in lung cancer cells was further detected by IHC, and the results showed that the expression of ATF-2 in NSCLC tissues was significantly higher than that in adjacent normal tissues and mainly concentrated in the nucleus (Figure 1(f)). The results showed that both ATF-2 and Wnt/Ca^2+^ signaling pathways were abnormally expressed in lung cancer cells, which may be related to the development of lung cancer.

### 3.2. Influence of ATF-2 Expression on Wnt/Ca^2+^ Signaling Pathway Activity and Proliferation of NSCLC

**Figure 2:**
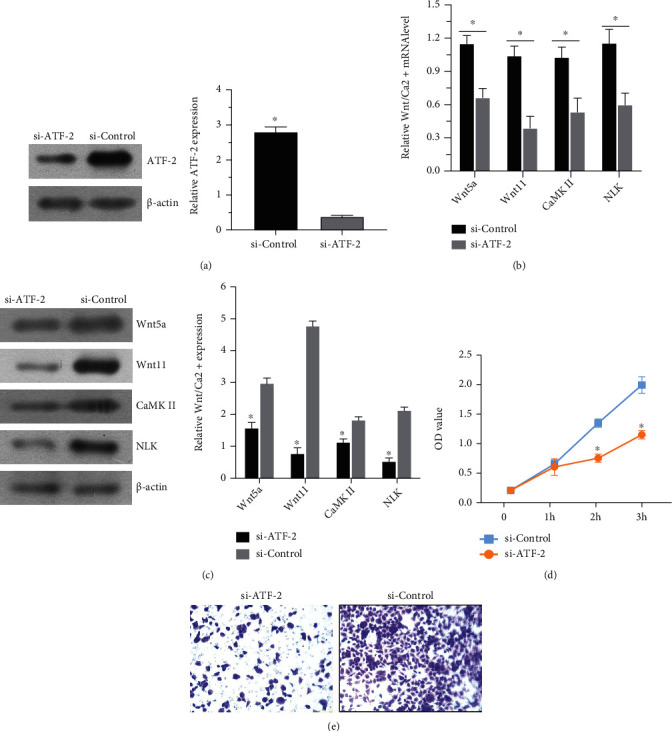
After knockdown of ATF-2, the effects of ATF-2 expression on the Wnt/Ca^2+^ signaling pathway and NSCLC proliferation and invasion were detected. (a) After ATF-2 knockdown, the expression of ATF-2 was detected by WB, and the knockdown efficiency was detected. (b, c) The activity of the Wnt pathway was detected by qPCR and WB. (d) CCK8 was used to detect cell proliferation. (e) Transwell tested the invasion ability of cells. Compared to the control group, ^∗^*P* < 0.05, the difference was statistically significant.

To investigate the effect of ATF-2 expression on Wnt/Ca^2+^ signaling pathway activity, we used CRISPR/Cas9 technology to knock out the ATF-2 gene in lung cancer cells. The WB method was used to detect the knockdown efficiency, and the results showed that ATF-2 gene knockdown significantly reduced the expression of ATF-2 (Figure 2(a)). Then, the expression of Wnt/Ca^2+^ pathway proteins was further detected in ATF-2 knockdown lung cancer cells. The results of qRT-PCR and WB assay showed that the expressions of Wnt5a and other proteins in the ATF-2 knockdown lung cancer cells (represented by si-ATF-2) were lower than those in the normal lung cancer cells (Figures 2(b) and 2(c)). After ATF-2 knockdown, Wnt/Ca^2+^ signaling pathway activity was decreased in lung cancer cells.

Then, the proliferation and invasion ability of si-ATF-2 cells was detected, and the proliferation and invasion ability of si-ATF-2 cells was significantly lower than that of normal lung cancer cells (Figures 2(d) and 2(e)). These results indicate that ATF-2 expression can affect the proliferation and invasion of NSCLC cells.

### 3.3. Wnt/Ca^2+^ Signaling Pathway and Its Influence on NSCLC Tissue

**Figure 3:**
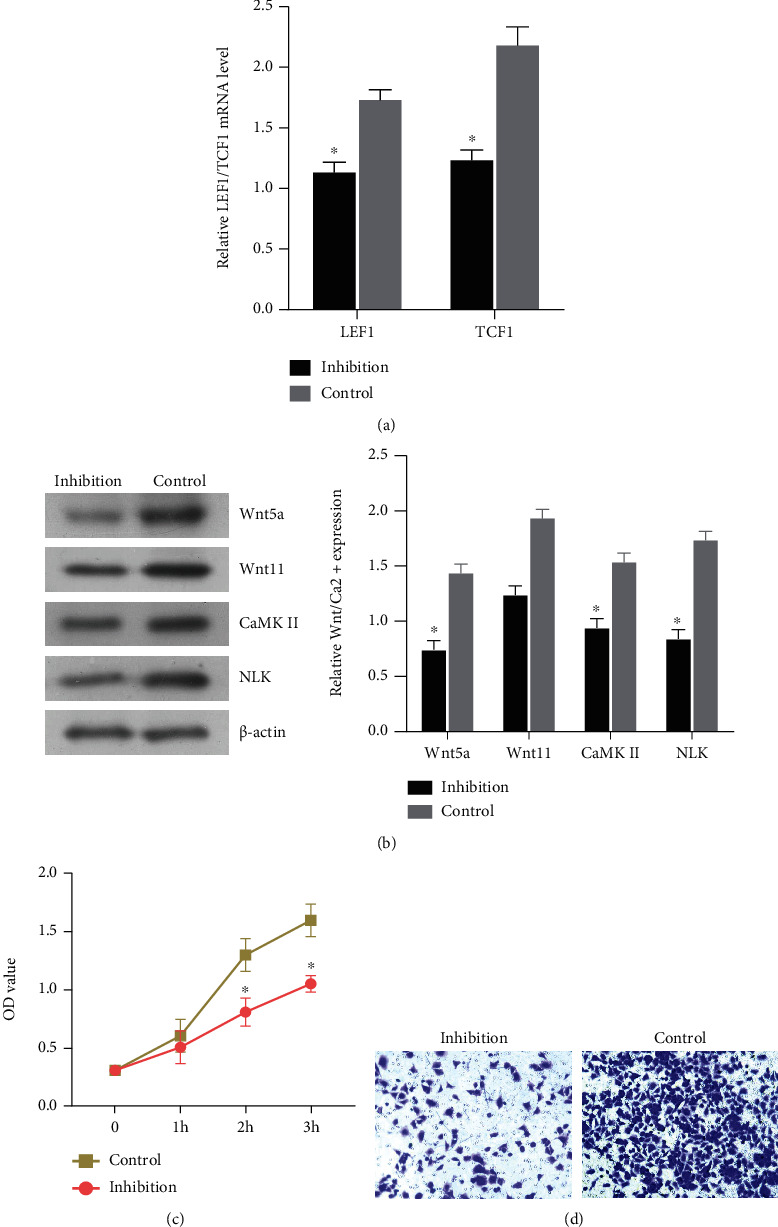
Wnt pathway inhibitors were added to detect the effect of Wnt/Ca^2+^ signaling pathway expression on NSCLC tissues. (a) The expression of the downstream gene LEF1/TCF1 was detected by qPCR to determine the inhibition efficiency. (b) The expression of Wnt pathway protein was detected by WB. (c) CCK8 was used to detect cell proliferation. (d) Transwell tested the invasion ability of cells. Compared to the control group, ^∗^*P* < 0.05, the difference was statistically significant.

In order to further study the effect of Wnt/Ca^2+^ signaling pathway activity on the proliferation and invasion ability of NSCLC cells, the pathway inhibitor (XAV939) was added into NSCLC cells to induce culture. RT-PCR was used to detect the expression of the downstream gene (LEF1/TCF1) in the signal pathway to determine whether the inhibitor had a successful effect. The expression of the transcription factor LEF1/TCF1 was significantly decreased in the inhibition group, and the inhibition-induced culture was successful (Figure 3(a)). Then, WB was used to detect the expression of pathway proteins Wnt5a, Wnt11, CaMK II, and NLK. The expression of Wnt5a and other proteins decreased significantly in the inhibition group, and the pathway activity decreased in the inhibition group (Figure 3(b)). CCK8 and Transwell were used to detect the ability of cell proliferation and invasion. The results showed that the proliferation and invasion ability of cells in the inhibition group decreased significantly compared with that in the control group (Figures 3(c) and 3(d)). After the addition of pathway inhibitors, the Wnt/Ca^2+^ signaling pathway activity was decreased, and cell proliferation and invasion ability was decreased.

### 3.4. ATF-2 Expression Activates the Wnt/Ca^2+^ Signaling Pathway and Promotes the Proliferation of NSCLC

**Figure 4:**
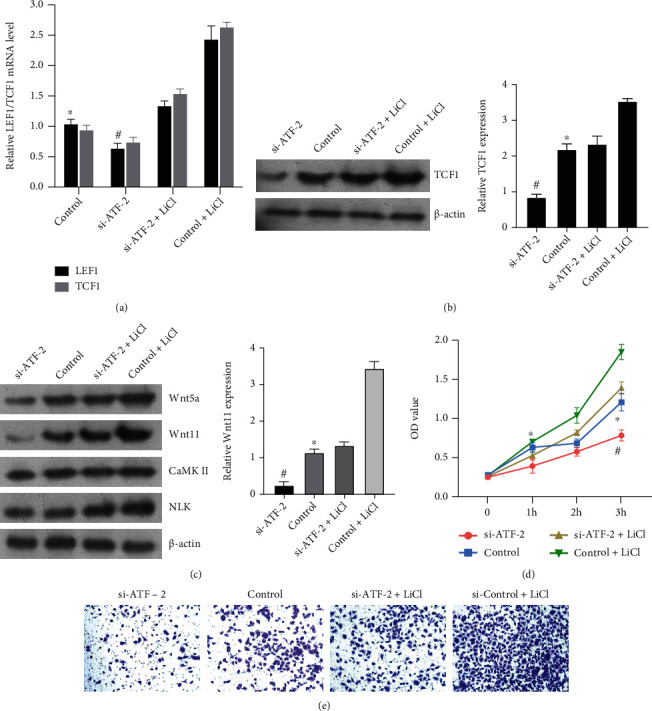
ATF-2 was knocked down in NSCLC cells, and the Wnt pathway agonist was added to detect pathway activity and cell proliferation activity. (a, b) qPCR and WB were used to detect the activity of downstream factors and determine the efficiency of agonists in the pathway. (c) WB was used to detect the expression of pathway proteins in different treatment groups. (d) CCK8 was used to detect cell proliferation. (e) Transwell tested the invasion ability of cells. Compared to the control group, ^∗^*P* < 0.05; compared to the si-ATF-2 group, ^#^*P* < 0.05; the difference was statistically significant.

To verify that ATF-2 expression can activate the Wnt/Ca^2+^ signaling pathway, CRISPR/Cas9 technology was used to knock out ATF-2, and a Wnt signaling pathway agonist (LiCl) was added to induce culture. The expression of the transcription factor LEF1/TCF1 was detected by RT-PCR and WB, and the expression of the transcription factor in the knockout group was the lowest; the expression of LEF1/TCF1 was the same as that of the control group in the cells induced by both ATF-2 gene knockout and pathway agonist. The expression of LEF1/TCF1 was the highest in the cells supplemented only with a pathway agonist (Figures 4(a) and 4(b)), and the pathway agonist-induced culture was successful. Then, WB was used to detect the expression of pathway proteins, and the results were the same as before. The expression of pathway proteins was the lowest after ATF-2 knockout. The expression of pathway proteins showed no significant difference compared with the normal control group after the knockout gene and the addition of the pathway agonist. The expression of pathway proteins was the highest in cells with only pathway agonist (Figure 4(c)). The results showed that the expression of the ATF-2 gene could activate the Wnt/Ca^2+^ signaling pathway.

CCK8 and other methods were used to detect the cells in the four groups. The cell proliferation and invasion ability of the ATF-2 gene knockout group was the lowest. After gene knockout and pathway agonist addition, cell proliferation and invasion ability was the same as that of the normal control group. The cells with only pathway agonists added had the highest proliferation and invasion abilities (Figures 4(d) and 4(e)). The results showed that ATF-2 could promote the proliferation and invasion of NSCLC by activating the Wnt/Ca^2+^ signaling pathway.

## 4. Discussion

Worldwide, lung cancer is the disease with the highest morbidity and mortality among malignant tumors, and studies on the pathogenesis and treatment methods of lung cancer are constantly updated [23]. ATF-2 is highly expressed in many cancer tissues and has been shown to promote the growth of cancer cells. For example, in pancreatic cancer, phosphorylated-ATF-2 protein expression is upregulated, promoting pancreatic cancer cell invasion and epithelial cell mesenchymal transformation [24]. miR-451 enhances drug resistance in renal cell carcinoma by targeting the expression of ATF-2 [25]. ATF-2 is known to be associated with the prognosis and survival of NSCLC patients and can promote the growth and development of NSCLC [19], but the mechanism remains unclear. Therefore, this paper mainly studies the mechanism by which ATF-2 expression affects the growth and development of NSCLC.

There are many signaling pathways that affect the proliferation of lung cancer cells, and the Wnt signaling pathway was selected in this paper. Among the Wnt signaling pathways, the Wnt/*β*-catenin pathway is the main pathway that affects the proliferation of lung cancer cells, so there are a lot of studies on the Wnt/*β*-catenin pathway. Yang et al. [26] have shown that FOXP3 promotes tumor growth and metastasis through activation of the Wnt/*β*-catenin signaling pathway. However, there are few studies on Wnt/Ca^2+^ Wnt signaling pathways, and some studies have shown that Wnt/Ca^2+^ signaling pathways are related to the expression of ATF-2. Therefore, this paper chose to investigate whether ATF-2 promotes the proliferation and invasion of NSCLC tissues through the Wnt/Ca^2+^ signaling pathway.

In this experiment, the expressions of ATF-2 and Wnt/Ca^2+^ signaling pathway proteins in NSCLC tissues were higher than those in other normal bronchial epithelial cells. After ATF-2 in A549 cells was knocked out with CRISPR/Cas9 technology, the expression of pathway proteins in A549 cells was significantly reduced, the activity of the Wnt/Ca^2+^ signaling pathway was decreased, and the proliferation and invasion ability of A549 cells was decreased. The results showed that ATF-2 expression could enhance the activity of the Wnt/Ca^2+^ signaling pathway and promote the proliferation and invasion ability of NSCLC cells. In order to verify the effect of the activity of the Wnt/Ca^2+^ signaling pathway on the proliferation and invasion ability of NSCLC cells, the Wnt pathway inhibitor (XAV939) was added into A549 cells to induce culture. The results showed that inhibition of pathway activity resulted in decreased gene expression and decreased proliferation and invasion ability of NSCLC cells. These results indicate that the expression of the Wnt/Ca^2+^ signaling pathway can promote the proliferation and invasion of NSCLC cells. Moreover, ATF-2 expression did not promote the Wnt/Ca^2+^ signaling pathway when it was inhibited. This suggests that ATF-2 expression needs to activate the Wnt/Ca^2+^ signaling pathway to play a promoting role. At last, the ATF-2 gene was knocked out or the agonist was added into A549 cells to induce culture. After the ATF-2 gene was knocked out, the expression of pathway genes decreased, and the proliferation and invasion ability of NSCLC cells decreased; after gene knockout and agonist addition, the expressions of Wnt5a and other proteins increased, and the proliferation and invasion ability of NSCLC cells also increased, which was similar to the blank control group. However, the cell proliferation and invasion ability of the agonist only was the highest. Therefore, ATF-2 may promote the proliferation and invasion ability of NSCLC cells by activating the Wnt/Ca^2+^ signaling pathway.

In summary, ATF-2 promotes cell proliferation and invasion by activating the Wnt/Ca^2+^ signaling pathway in NSCLC tissues. It proves the application of the Wnt/Ca^2+^ signaling pathway in NSCLC tissue and provides more ideas for the study of the Wnt/Ca^2+^ signaling pathway in lung cancer.

## Data Availability

The datasets used and/or analyzed during the current study are available from the corresponding author on reasonable request.
